# First Principles Prediction of Topological Phases in Thin Films of Pyrochlore Iridates

**DOI:** 10.1038/srep11072

**Published:** 2015-06-16

**Authors:** Xiang Hu, Zhicheng Zhong, Gregory A. Fiete

**Affiliations:** 1 Department of Physics, The University of Texas at Austin, Austin, TX 78712, USA; 2 Institute of Solid State Physics, Vienna University of Technology, A-1040 Vienna, Austria

## Abstract

While the theoretical and experimental study of topological phases of matter has experienced rapid growth over the last few years, there remain a relatively small number of material classes that have been experimentally shown to host these phases. Most of these materials contain bismuth, and none so far are oxides. In this work we make materials-specific predictions for topological phases using density functional theory combined with Hartree-Fock theory that includes the full orbital structure of the relevant iridium *d*-orbitals and the strong but finite spin-orbit coupling strength. We find Y_2_Ir_2_O_7_ bilayer and trilayer films grown along the [111] direction can support topological metallic phases with a direct gap of up to 0.05 eV, which could potentially bring transition metal oxides to the fore as a new class of topological materials with potential applications in oxide electronics.

Topological phases have attracted much interest in recent years^1^,^2^,^3^. While there are a number of three-dimensional materials exhibiting topological properties^4^, there are relatively few two-dimensional examples^5^,^6^,^7^ aside from the well-known quantum Hall systems^8^. Among the known three-dimensional topological insulators, there remains the persistent problems of high bulk conductivity and surface state properties that can change significantly over the course of a few hours or days^4^. Therefore, it is desirable to seek out new material classes that might support topological phases, including those that may have a metallic character^9^,^10^,^11^,^12^. This paper reports theoretical research directed at the goal of finding new material classes that do not yet have experimentally verified topological states.

Transition metal oxides (TMO) with heavy 4*d* or 5*d* transition metal ions, such as iridium, have drawn considerable experimental and theoretical interest in this regard^13^, and may also form a natural connection to the field of oxide electronics^14^,^15^,^16^. Particularly on the theoretical side, the pyrochlore iridates have motivated a number of studies predicting novel topological phases in three dimensions^17^,^18^,^19^,^20^,^21^,^22^,^23^. However, it is now appreciated that three-dimensional pyrochlore iridates of the form *R*_2_Ir_2_O_7_, where *R* is a rare-earth element such as La, Y, or Eu may not have the correct electronic band structure features to support topological insulating states—namely that there is a (4-fold) band degeneracy at the Fermi energy protected by crystal symmetry^13^,^22^,^24^. This symmetry-protected gapless point at the Fermi energy precludes insulating states in the absence of crystal symmetry breaking and therefore strongly disfavors the proposals^17^,^18^,^19^,^20^,^21^,^22^,^23^ in this class of materials.

On the other hand, there is no direct connection between thin-film band structure and bulk band structure because films can have different thicknesses and be grown along different crystalline directions, both of which have strong effects on the energy bands. Thus, in addition to bulk TMO as candidates for supporting three-dimensional topological phases, several thin-film TMO systems have also been suggested as potential hosts for two-dimensional topological phases^25^,^26^,^27^,^28^,^29^,^30^,^31^,^32^,^33^,^34^,^35^,^36^,^37^. A short review of recent theoretical work on topological phases in transition metal oxide films is now available^38^. Besides the integer and fractional quantum Hall effects that arise from the application of a magnetic field perpendicular to a two-dimensional electron gas at low-temperature^8^, two other important topological states in two-dimensions are the time-reversal invariant *Z*_2_ topological insulators^39^,^40^,^41^,^42^ (TI) and the zero magnetic field, time-reversal symmetry broken Chen insulator (CI) phase^43^. In a Chern insulator, interactions drive a spontaneous magnetization that leads to an effective magnetic field acting on the electrons, so an external magnetic field is not necessary. This “internal” magnetic field is such that the electron bands in the presence of the magnetization are gapped around the Fermi energy and have a quantized Chern number^25^,^26^,^44^, and therefore a corresponding quantized Hall effect in zero external magnetic field. The quantum Hall effects, the *Z*_2_ TI, and the CI all share the property that they are electrical insulators in the bulk but have topologically protected gapless edge states. The one-dimensional edge states of these topological systems are most clearly revealed in electrical transport measurements^5^,^6^,^7^.

In this work, we use first-principles density functional theory (DFT) calculations combined with Hartree-Fock theory to show that transition metal oxide films can support “topological metals” of the time-reversal invariant variety as well as time-reversal symmetry broken “Chern metals”. The “topological metals” we find are quite similar in their band structure to antimony^45^: The direct gap remains open throughout the Brillouin zone (BZ), but the indirect gap is negative so that the materials are semimetals. Because of the direct gap throughout the BZ, it remains possible to define a topological invariant^45^, though the strict topological protection of the edges are lost. Nevertheless, if the system is relatively clean, the edge states will retain properties qualitatively similar to the corresponding insulating partner (either *Z*_2_ TI or CI) that would be obtained if the band structure were “deformed” in such a way to make the indirect gap positive while maintaining the positive direct gap.

In thin-film TMO, both the time-reversal invariant *Z*_2_ TI^25^,^26^,^29^,^34^,^35^,^37^ and the time-reversal symmetry broken CI phase (sometimes also referred to as a quantum anomalous Hall state)^25^,^26^,^27^,^29^,^31^,^32^,^33^,^36^,^37^ have been predicted. In real material systems, both the two-dimensional TI and CI states rely on spin-orbit coupling. In the context of thin-film TMO, the spin-orbit coupling may be “dynamically generated” through interactions for light transition metals^25^,^26^, or may be intrinsic for oxides with heavy transition metal ions^37^. In this work, we use a combination of first-principles DFT calculations and Hartree-Fock theory to show bilayer and trilayer films of Y_2_Ir_2_O_7_ grown along the [111] direction can support topological metallic phases^9^,^10^ (in the sense described above) with a direct gap of up to 0.05 eV. Under the right perturbations (which can include substrate strain and charge density wave order), these phases can be driven to their insulating topological counterparts.

## Results

**Figure 1:**
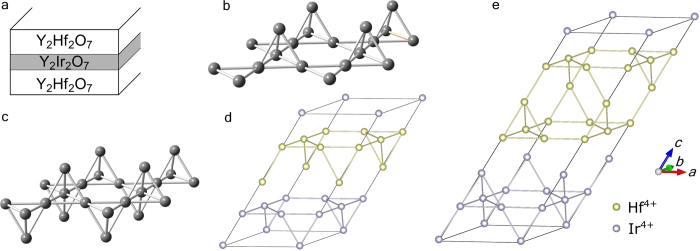
Thin film geometry. (**a**) “Sandwich” structure of system. (**b**) [111] grown bilayer lattice structure of Ir^4+^ ions in Y_2_Ir_2_O_7_. (**c**) [111] grown trilayer lattice structure of Ir^4+^ ions in Y_2_Ir_2_O_7_. Supercells used for bilayer (**d**) and trilayer (**e**) systems in first-principles density functional theory calculations.

We study a sandwich structure consisting of a thin (few atomic layers) Y_2_Ir_2_O_7_ film between the non-magnetic, large-gap band insulator Y_2_Hf_2_O_7_, as shown in Fig. 1(a). We find quantitatively similar results if La_2_Ir_2_O_7_ replaces Y_2_Ir_2_O_7_. The “capping layers” of Y_2_Hf_2_O_7_ serve mainly to stabilize the thin Y_2_Ir_2_O_7_ films, but also strain the films and modify their band structure compared to the unstrained case. In our study, we used first-principles DFT calculations (see Methods) to determine the band structure of the system, and included different thicknesses of Y_2_Hf_2_O_7_ in fully relaxed structures [for a fixed Y_2_Ir_2_O_7_ film, either bilayer, Fig. 1(b), or trilayer, Fig. 1(c)] to assess the magnitude of the strain effects. The supercells we used in our first-principles calculations are shown in Fig. 1(d,e). Our calculations show the strain from the lattice mismatch (1.9%) between Y_2_Ir_2_O_7_ and Y_2_Hf_2_O_7_ is not a large effect in this system (possibly because of the spatially extended Ir *d*-orbitals), and therefore we do not believe the choice of Y_2_Hf_2_O_7_ is integral to the physics of the Y_2_Ir_2_O_7_ films. Other choices of wide-gap band insulator “capping layers” could be made to investigate strain effects in more detail.

**Figure 2:**
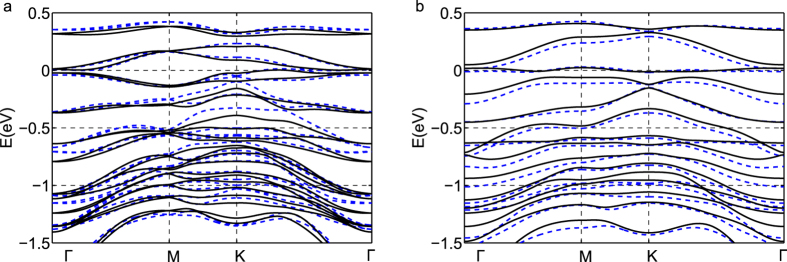
Electronic band structure for Y_2_Ir_2_O_7_ thin films grown along [111] with the supercells shown in Fig. 1. Bilayer (**a**) and trilayer (**b**) band structure from a fully relaxed GGA + SOC calculation [solid black] with Wannier fit [dashed blue]. The Wannier fits (obtained from slab-truncation of bulk Wannier projection) are used in the Hartree-Fock calculation of the phase diagrams of the bilayer and trilayer.

The results of our density functional theory calculations for the structures in Fig. 1 are shown in Fig. 2 where the energy bands are plotted along high-symmetry directions in the first BZ. In our calculations we have imposed a non-magnetic solution. Since the energy bands cross the Fermi energy (set to zero in Fig. 2), our results indicate that both the bilayer and trilayer systems are metallic in the absence of strong correlations. We note that since the bilayer breaks inversion symmetry, the bands in Fig. 2(a) are non-degenerate, while the bands of the inversion symmetry-preserving TKT layer in Fig. 2(b) are two-fold degenerate. The larger spatial extent of the 5*d*-orbitals compared to the 3*d*-orbitals is expected to make TMO films with heavy transition metals less susceptible to interfacial strain—a quality that might enhance the robustness of theoretical predictions for topological phases in thin-film systems with heavier elements. The dashed blue lines are Wannier fits to the band structure that are used in further Hartree-Fock calculations to better account for correlation effects. We now describe these Hartree-Fock calculations.

After obtaining a *non-interacting* Hamiltonian in a local (*t*_2*g*_-like Wannier) basis for the bilayer and TKT trilayer (the dashed blue lines shown in Fig. 2),
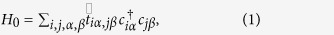


we studied the full Hamiltonian *H* = *H*_0_ + *H*_*U*_ where
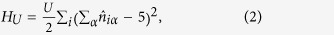
within Hartree-Fock theory. In Eq.(1) and Eq.(2), *i*,*j* represent different sites, *α*,*β* indexes different orbitals (including spin indicies) in the *t*_2*g*_ manifold, and 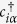 (*c*_*iα*_) is the creation (annihilation) operator of an electron on site *i* with orbital *α*. The complex hopping amplitude 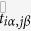 includes spin-orbit coupling, and are obtained from the Wannier fit to the DFT results. The rotationally invariant (in orbital and spin space) Hubbard term, Eq.(2), follows the convention used in previous works^18^, and 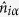 is the number operator for site *i*, orbital *α*. The rotationally invariant form allows the Hartree-Fock calculation to be performed in any basis, and allows us to extend most previous studies on the iridates by explicitly including the full *t*_2*g*_ subspace and keeping the spin-orbit coupling finite, as opposed to working in a strong (infinite) spin-orbit coupling limit with only *j* = 1/2 states in the Hilbert space. The main approximation made in the form of the interaction given in Eq.(2) is the neglect of the Hund’s coupling. Physically, this restricts our results to low-spin configurations on the iridium ions, which is consistent with prior work^22^,^46^.

**Figure 3:**
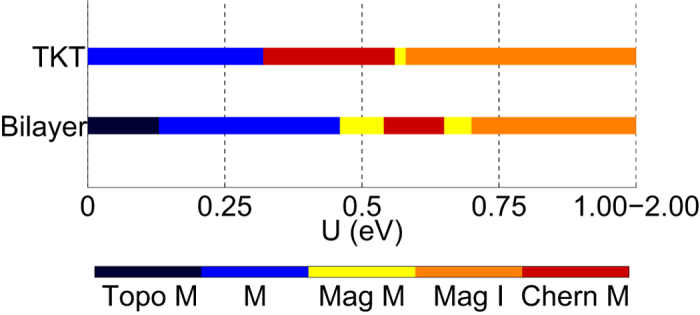
Hartree-Fock phase diagram of the bilayer and TKT thin films. For small *U* the bilayer exhibits a topological metal phase with time-reversal symmetry preserved. For a somewhat larger *U*, time-reversal symmetry is spontaneously broken for both the bilayer and trilayer, with Chern metals appearing in the bilayer and TKT films centered around *U* ≈ 0.6 eV ad *U* ≈ 0.5 eV respectively. If disorder in the system is not too strong, edge modes in the topological metal and Chern metals can behave qualitatively similar to their insulating counterparts. Topo M = topological metal, M = metal, Mag M = magnetic metal, Mag I = magnetic insulator, and Chern M = Chern Metal.

The Hartree-Fock calculation is performed by decoupling the on-site interactions, Eq.(2), as
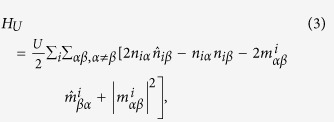
where 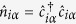, and 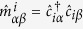 with *α* ≠ *β*. We perform an unrestricted Hartree-Fock calculation in which the filling in the unit cell is held fixed while the electron density on each site is allowed to vary. The self-consistent calculation generates a local minimum of the total energy through the variation of the coupling constants 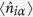 and 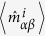, which are determined by the eigenstates below the Fermi level. *Z*_2_ and Chern invariants are calculated as described in the supplementary material. The Hartree-Fock results for the [111]-grown bilayer and TKT trilayer are shown in Fig. 3, where the strength of *U* is varied. Because *U* is an effective interaction parameter within the *t*_2*g*_ manifold, it is difficult to determine its precise value from experiment or theory. Estimates in the literature range from 0.4–2.0 eV^13^, so we include this range in our calculations.

**Figure 4:**
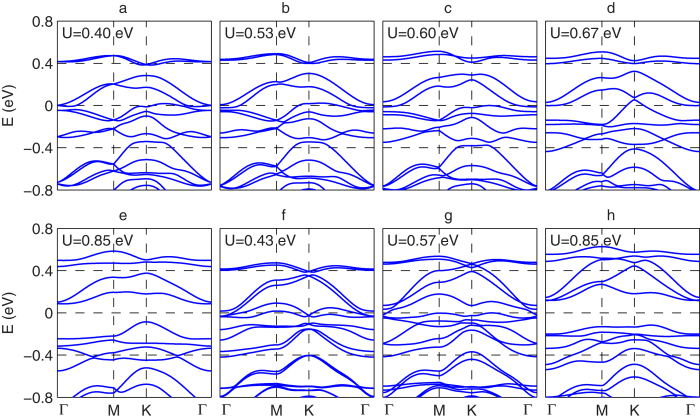
Bilayer and trilayer (TKT) electronic band structure for the different phases shown in Fig. 3 as a function of interaction parameter U in Eq.(2). From (**a**–**e**) the bilayer thin film undergoes M → Mag M → Chern M → Mag M → Mag I. From (**f**–**h**), the TKT thin film undergoes Chern M → Mag M → Mag I. The Fermi energy for each plot has been set to zero. The Topo M phase in bilayer and M phase in TKT thin films have band structure similar to Fig. 2, so they are not shown here.

Based on the band structures shown in Fig. 2, the small-*U* behavior is expected to be metallic, and we indeed find this is the case. From Fig. 2(a,b) one can see that the band width of the states closest to the Fermi energy are on the order of 0.5 eV. Thus, one expects possible transitions when *U* is of the order of 0.5 eV. Indeed, one finds that in both systems time-reversal symmetry in spontaneously broken (*i.e.*, magnetism sets in) for interactions of this rough magnitude. By investigating the small *U* metallic phases in both cases, we find that the metallic state in the bilayer is actually a topological metal^2^: While the direct gap is finite throughout the Brillouin zone, the indirect gap is negative. The finite direct gap allows one to compute the *Z*_2_ invariant^47^ for the lowest 20 bands, and we find that it is topologically non-trivial. Therefore, if one were able to deform the bands so that the direct gap remains open while the indirect gap is made positive, one would obtain a *Z*_2_ topological insulator. Because the change in energy of states near the Fermi energy required to do this is of the order of a few tens of meV, a judicious choice of substrate in experiment may in fact turn the bilayer system into a *Z*_2_ topological insulator if interactions are sufficiently screened with a nearby metallic gate so that the effective *U* value is reduced compared to the bulk value. A closer examination of magnetic cases also reveals a topological metallic phase, the Chern metal around *U* ≈ 0.6 eV. The situation in this case is qualitatively similar to the *Z*_2_ topological metal, except that the Chern metal has broken time-reversal symmetry: The Chern metal has a finite direct gap throughout the Brillouin zone, but a negative indirect gap such that the lowest bands have a total Chern number^48^ of 1 so that a deformation of the Hamiltonian that keeps the direct gap open but makes the indirect gap positive would result in a Chern insulator. Representative band structures for the bilayer for the four different phases found are shown in Fig. 4(a–e).

**Figure 5:**
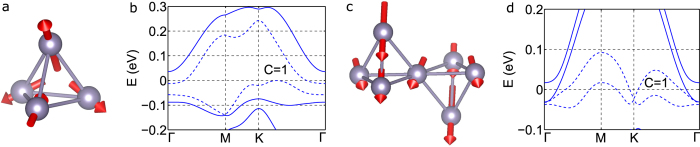
The electronic band structure and local magnetic moments of the Chern metal phase in the bilayer and TKT thin films. (**a**) Orientation of the local magnetic moments in the bilayer thin film when *U* = 0.60 eV. (**b**) A finer resolution of the electronic band structure of the Chern metal (*U* = 0.60 eV in the bilayer thin film) close to the Fermi energy, which we have set to zero. (**c**) Orientation of the local magnetic moments in the TKT thin film when *U* = 0.43 eV. (**d**) A finer resolution of the electronic band structure of the Chern metal (*U* = 0.43 eV in the TKT thin film) close to the Fermi energy, which we have set to zero. The dash lines in (**b**) and (**d**) represent the valence and conducting bands. The bands below the direct gap on each plot possess a total Chern number equal to one.

Similar to the bilayer case, the TKT trilayer thin film also reveals a Chern metal around *U* ≈ 0.5 eV. Representative band structures for the trilayer for the three different phases found shown in Fig. 4(f–h). In contrast to the results for the bulk system (see Discussion and supplementary material), in both bilayer and TKT thin films we do not find an “all-in/all-out” magnetic order, but instead a deformation of it (shown in Fig. 5) that has a net magnetic moment.

In summary, we have reported a combined first-principles DFT and Hartree-Fock study of bilayer and trilayer films of Y_2_Ir_2_O_7_. Using realistic electronic band structure with a finite spin-orbit coupling included, we calculate the effect of interactions within the full *t*_2*g*_ iridium 5*d*-orbital subspace and find the thin films systems may support topological metallic phases with the properties described earlier. These topological metallic phases could be converted into their insulating counterparts with the right substrate strain or charge density wave order. We hope this work will help encourage further experimental efforts in this direction and facilitate the discovery of topological phases in transition metal oxides.

## Discussion

In order to cross-check our results and theoretical techniques, we initially performed first principles electronic structure calculations (see Methods) on the *bulk* Y_2_Ir_2_O_7_ system, for which prior results are available^22^. This check highlighted a number of important points of physical significance. First, a Hartree-Fock calculation on a Hamiltonian of the form in Eq.(1) and Eq.(2) for bulk Y_2_Ir_2_O_7_ results in the same “all-in/all-out” magnetic state reported earlier^22^ for a moderate Hubbard *U* value of 0.7–1.8 eV. This establishes that our approach and methods can capture the important details obtained in earlier calculations for the bulk systems. Second, the quality of the Wannier fit to the bulk band structure obtained from first-principles (see supplementary material) are in fairly good agreement, including important details such as the degeneracies at the Γ point for bands near the Fermi energy (set equal to zero). In addition, the upper 4 bands (roughly, the total angular momentum *j* = 1/2 states) and the lower 8 bands (roughly, the total angular momentum *j* = 3/2 states) are *not* well separated from each other. This implies that the strong spin-orbit coupling limit in which the *j* = 1/2 manifold is well separated from the *j* = 3/2 manifold is not realized in this material. For this reason, we used the non-magnetic DFT result (without a “*U*”) as input for a Hartree-Fock calculation that includes interactions within the *full* 5*d t*_2*g*_-orbital manifold. Third, the thin film tight-binding models are obtained by truncating the bulk Wannier-fit. Compared with the bulk electronic band structure, the quality of the fit is less good for the films. (See supplementary for details.) We attribute this to the lower symmetry of the films compared to the bulk and the fact that the iridium 5*d*-orbitals are rather extended. As we show in the supplementary information, a *tight-binding fit* to the bulk band structure that includes out to third neighbor hopping yields a rather poor fit to the DFT results. As a result, the iridium 5*d*-orbitals in the bilayer and trilayer films have a spatial extent that is at least comparable to the film thickness and therefore the 5*d*-orbitals are influenced by the “capping” Y_2_Hf_2_O_7_ layers and experience a local environment of reduced symmetry. While this leads to a slightly less good Wannier fit than we obtained for the bulk, the bands and the density of states around the Fermi energy are reasonably close. Therefore, we expect there to be little numerical difference in a Hartree-Fock calculation that makes use of the Wannier fit shown in Fig. 2, and one that makes use of a fit that better approximates the DFT result. Fourth, an important corollary to the more extended orbitals of 5*d* TMO is that thin films should be less sensitive to substrate strain than 3*d* elements; this may enhance their stability in device applications.

## Methods

Our first-principles density functional theory (DFT) calculations were carried out in the generalized gradient approximation (GGA) of the exchange-correlation potential with a 10 × 10 × 10 k-point grid. The computations were performed in two different ways (see supplementary material): (i) By using the all-electron full-potential augmented plane-wave method in the WIEN2k implementation, and (ii) By using a norm-conserving pseudopotential in the Quantum Espresso implementation. The two different packages obtain very close results. The spin-orbit coupling was included in the fully-relativistic schemes, and included non-zero spin-orbit coupling on all the atoms. We took the experimental structure of bulk Y_2_Ir_2_O_7_ with space group (227, Fd-3m). Each unit cell contains four equivalent Ir^4+^ sites. In this material, each oxygen octahedral cage is subjected to a trigonal distortion, which splits the *t*_2*g*_ orbitals into *e*'_*g*_ and *a*_1*g*_ states^49^. The Wannier projections (see supplementary material) including spin-orbit coupling were carried out with a 8 ×8 ×8 mesh size with an initial basis of the local *t*_2*g*_ orbitals at each Ir^4+^ site with the spin-quantization axis set to the global z-axis. The thin film DFT calculations (see supplementary material) were carried out in the (Y_2_Ir_2_O_7_)_2_/(Y_2_Hf_2_O_7_)_2_ and (Y_2_Ir_2_O_7_)_3_/(Y_2_Hf_2_O_7_)_3_ superlattices, whose structure was fully relaxed. All the Hartree-Fock calculations in the thin films were unrestricted with self-consistency obtained from randomly generated initial parameters.

## Additional Information

**How to cite this article**: Hu, X. *et al.* First Principles Prediction of Topological Phases in Thin Films of Pyrochlore Iridates. *Sci. Rep.* **5**, 11072; doi: 10.1038/srep11072 (2015).

## Supplementary Material

Supplementary Information
