# Supplementary material for: First Principles Prediction of Topological Phases in Thin Films of Pyrochlore Iridates
Source: Sci Rep. 2015 Jun 16;5:11072. doi: 10.1038/srep11072 (PMC4468522; doi:10.1038/srep11072)
Supplement: Supplementary Information [file srep11072-s1.pdf]

Supplemental Materials for "First Principles Prediction of Topological Phases in Thin Films of Pyrochlore Iridates"

Xiang Hu,[1] Zhicheng Zhong,[2] and Gregory A. Fiete[1]

[1]*Department of Physics, The University of Texas at Austin, Austin, Texas 78712*
[2]*Institute of Solid State Physics, Vienna University of Technology, A-1040 Vienna, Austria*

(Dated: March 25, 2015)

CONTENTS

I. Bulk DFT calculation for $Y_2Ir_2O_7$ 2

II. Tight-binding fit to the bulk DFT calculation 2

III. Wannier projection of the bulk DFT calculation for $Y_2Ir_2O_7$ 3

IV. Details of thin film DFT calculation for $(Y_2Ir_2O_7)_n/(Y_2Hf_2O_7)_m$ 3

V. Thin film Hartree-Fock calculation 5

References 6

I. BULK DFT CALCULATION FOR $Y_2Ir_2O_7$

The DFT calculation of bulk $Y_2Ir_2O_7$ was carried out with `WIEN2k`[1] and `Quantum Espresso` (QE)[2]. The results obtained from these two codes agree well. In QE, the pseudopotentials are generated by the included `ATOMIC` code. In the pseudopotential generation we used the fully relativistic PBESOL functional[3]. Because QE can only accept norm-conserving pseudopotentials for finite spin-orbit coupling (SOC) when Wannier projection is desired, we took all pseudopotentials to be norm-conserving. The valence shell of Y ([Kr]$4d^1 5s^2$) includes the $4s, 4p, 4d, 5s, 5p$ orbitals, and the valence shell of Ir ([Xe]$4f^{14}5d^7 6s^2$) includes $5s, 5p, 5d, 6s$, and $6p$ orbitals. Including the semi-core states, that is, $4s, 4p$ in Y, and $5s, 5p$ in Ir improves the transferability of the pseudopotentials. The valence states of O ([He]$2s^2 2p^4$) include the $2s$ and $2p$ states. The cutoff energy in QE calculation was selected to be 150 Rydberg (Ry) for plane waves, and 600 Ry for the charge densities. The structure information of bulk $Y_2Ir_2O_7$ is from Ref.[4]. The positions of the iridium ions in the bilayer and trilayer films grown along [111] are shown in Fig.S1.

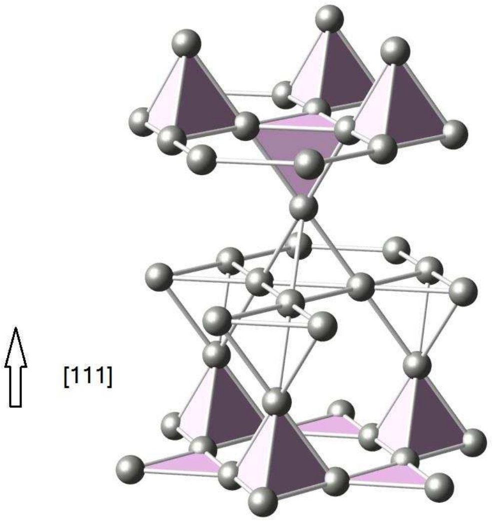

FIG. S1. Positions of iridium ions in the [111] grown bilayer (bottom) and trilayer (top) structure.

II. TIGHT-BINDING FIT TO THE BULK DFT CALCULATION

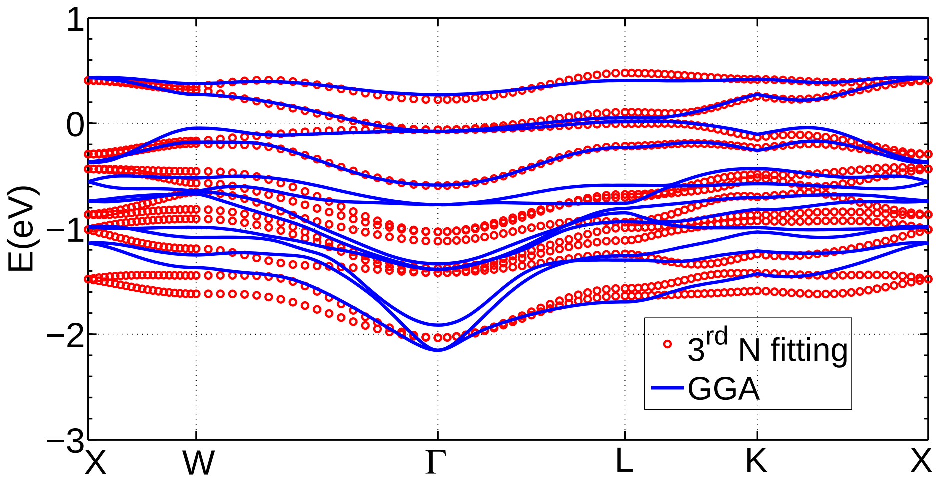

FIG. S2. Tight binding fit with up to the 3rd neighbor hopping compared to a GGA calculation in `WIEN2k`.

Because the tight-binding fitting method has been successfully applied to period four transition metal oxides, such as $LaNiO_3$[5,6], we first tried to fit the DFT results for bulk $Y_2Ir_2O_7$ to a tight binding model that included both direct and indirect hopping between the local t_{2g} orbitals of the Ir^{4+} ions. The hopping matrix elements were generated using Slater-Koster (SK)[7] integrals. As shown in Fig.S2 above, in order to obtain even a semi-quantitative fit for the $j = 1/2$ bands (upper 4) one must include at least out to third-neighbor hoppings. Even in this case, however, the lower $j = 3/2$ bands are fit rather poorly. The failure of the tight-binding fitting reveals the complexity of $5d$ orbits, and their much larger spatial extent compared to the $3d$ orbitals. In order to obtain an satisfactory model, one must include more SK hopping terms in the tight-binding fitting, which is not well motivated for a practical model Hamiltonian. Instead, we turn to the Wannier states, making use of the `Wannier90` package[8], which yields the fit shown in Fig.S3.

III. WANNIER PROJECTION OF THE BULK DFT CALCULATION FOR $Y_2Ir_2O_7$

The spin-orbit coupling can be treated in two different ways based on Wannier projection. One route is to first perform a DFT calculation and Wannier projection without SOC, and then add an onsite SOC ($\sim \lambda(-\mathbf{l}) \cdot \mathbf{s}$) term "by hand" whose strength is determined through least-square fitting of the Wannier+SOC bands to the DFT bands with the SOC *turned on*[8,9]. Alternatively, one can perform a DFT calculation with SOC turned on and then obtain a direct Wannier projection of the SOC coupled states[8].

In those Wannier projections, the initial basis is selected as the *local* t_{2g} orbitals. Even though the trigonal distortion causes a mixing between those t_{2g} orbitals, the axis of the local t_{2g} orbitals on each of the 4 sites in the unit cell can be assigned as[8]

$$\begin{aligned} x_1 &= (2/3, -1/3, 2/3), \\ z_1 &= (-2/3, -2/3, 1/3); \\ x_2 &= (2/3, -2/3, 1/3), \\ z_2 &= (1/3, 2/3, 2/3); \\ x_3 &= (1/3, 2/3, -2/3), \\ z_3 &= (2/3, 1/3, 2/3); \\ x_4 &= (1/3, -2/3, 2/3), \\ z_4 &= (2/3, -1/3, -2/3). \end{aligned} \tag{S1}$$

The coordinates in Eqs.(S1) can be easily obtained from the rotational matrices $R^{(0)-(3)}$ in Ref.[10]. All the Wannier functions are centered on the iridium ions. The spin quantization axis for all 4 iridium ions in the unit cell is set to the the global z-axis. To maximumly preserve the bulk crystal symmetry, the Num_Iter is set to 0 in the Wannierization process. However, even if a finite Num_Iter is used, the Wannier functions change only slightly, so the trial wave functions are quite good.

When the SOC is fit "by hand", we find a spin-orbit coupling strength of about 0.43 eV, corresponding roughly to the splitting of the $j = 1/2$ and $j = 3/2$ manifolds. As shown in Fig.S3, this leads to a reasonable fit to the fully relativistic (SOC on) DFT calculation. This approach should be able to capture the main character of the phases when interactions are further treated at the Hartree-Fock level. By comparison, a direct Wannier projection for the DFT calculation with SOC turned on captures the band feature more accurately (the blue dashed line in Fig.S3) and will be more accurate in determining the phase diagrams.

From the analysis of the Wannier projection ones sees appreciable hopping between iridium ions that are as far as 3 to 4 FCC basis vectors from each other. Our results agree with a previous study in Sr_2IrO_4, in which the hopping between Ir^{4+} ions 1 nm (around 3 times the distance between our nearest neighbor hopping) away from each other still plays a role[11].

IV. DETAILS OF THIN FILM DFT CALCULATION FOR $(Y_2IR_2O_7)_n/(Y_2HF_2O_7)_m$

In order to carry out our the thin film calculations, we construct the superlattice with $Y_2Ir_2O_7$ sandwiched between the band insulator $Y_2Hf_2O_7$[12]. We consider a superlattice $(Y_2Ir_2O_7)_n/(Y_2Hf_2O_7)_m$, where the sub-indexes n and m represent the numbers of Ir^{4+} or Hf^{4+} layers. To investigate the effect of strain, other substrates, such as $Y_2Ti_2O_7$[12] can also be used.

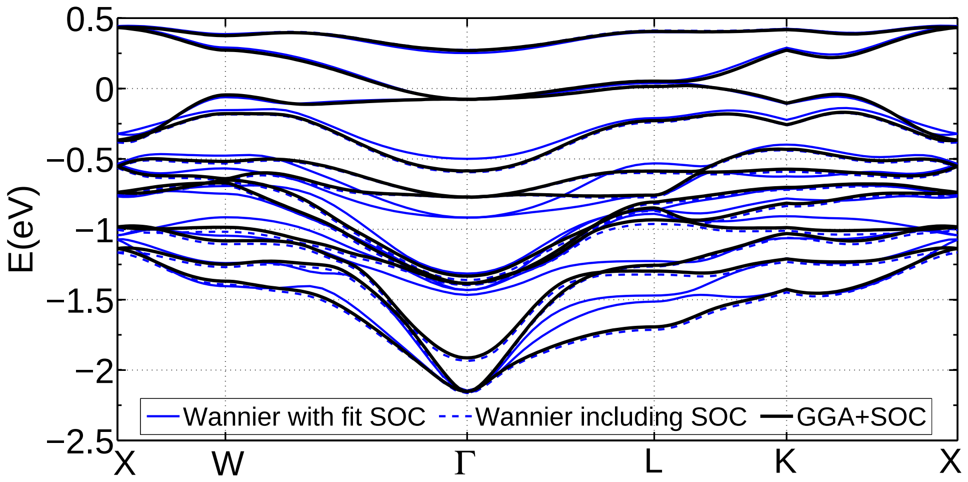

FIG. S3. A comparison of the two methods used to treat the spin-orbit coupling (SOC). The black curves are the band structure from the DFT calculation with SOC turned on for all atoms. The blue solid curves show the band structure obtained from the TB model. The TB model was obtained from Wannier projection of the DFT calculation with SOC turned off supplemented with a SOC added on "by hand" and fit to the DFT band structure with the SOC turned on. The fit value of the SOC is 0.43eV. The dotted curves show the band structure resulting from the direct Wannier projection of the DFT calculation with SOC turned on. The DFT results were obtained through `WIEN2k`. All the Wannier projections were done in `QE+Wannier90`.

The original FCC basis vectors for the bulk pyrochlore lattice are given by,

$$\mathbf{a}_1 = (0, a/2, a/2),$$
$$\mathbf{a}_2 = (a/2, 0, a/2),$$
$$\mathbf{a}_3 = (a/2, a/2, 0),$$

where a is the lattice constant.

To obtain the thin film structure along [111] direction, for the $n = 2, m = 2$ bilayer thin film, the basis vectors are selected as

$$\mathbf{a} = \mathbf{a}_3 - \mathbf{a}_1,$$
$$\mathbf{b} = \mathbf{a}_2 - \mathbf{a}_1,$$
$$\mathbf{c} = -2 * \mathbf{a}_1.$$

Here $\mathbf{a}$ and $\mathbf{b}$ are in plane, while $\mathbf{c}$ contains a component out of the plane spanned by $\mathbf{a}$ and $\mathbf{b}$. The position of each ion can be expressed in terms of linear combinations of $\mathbf{a}, \mathbf{b}, \mathbf{c}$. The size of the unit cell has been doubled compared to the bulk, with Ir^{4+} ions in two adjacent atomic layers substituted with Hf^{4+} ions to obtain the unrelaxed bilayer structure shown in Fig.S4. As an initial guess, the lattice constant a can be taken as the average of $Y_2Hf_2O_7$ and $Y_2Ir_2O_7$. The unrelaxed trilayer film structure can be obtained in a similar way. Some package such as `ASE Surface`[13] can also help to achieve this.

The determination of the lattice structure of the sandwhich is carried out in a fully relaxed scheme with scalar-relativistic[14] pseudopotentials for both the bilayer superlattice $(Y_2Ir_2O_7)_2/(Y_2Hf_2O_7)_2$ and the trilayer superlattice $(Y_2Ir_2O_7)_3/(Y_2Hf_2O_7)_3$. We have verified that increasing the number of layers of $Y_2Hf_2O_7$ does not have a significant effect on the final lattice structures and the resulting band structures. Once the lattice structure is determined, the band structures of the bilayer and trilayer $Y_2Ir_2O_7$ are calculated in a fully relativistic basis (*i.e.*, with the spin-orbit coupling turned on). The pseudopotential of Hf (with atomic configuration $[\mathrm{Xe}]4f^{14}5d^26s^2$) is generated in the same way as the other elements, with the valence orbits as $5s, 5p, 5d, 6s$, and $6p$. The k-space is meshed by $7{\times}7{\times}1$ with Monkhost-Pack Grid[15]. The Wannier fits shown in Fig. 2 of the main text are based on the Wannier fits obtained for the *bulk* system, which are still fairly accurate for states near the Fermi energy.

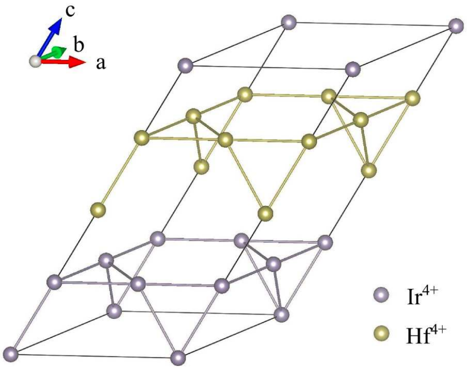

FIG. S4. The unrelaxed bilayer thin film. Only the Ir^{4+} and Hf^{4+} ions are displayed.

V. THIN FILM HARTREE-FOCK CALCULATION

The unrestricted Hartree-Fock calculation is carried out in the *full* t_{2g} subspace of the iridium orbitals with Wannier states obtained from the bulk band structure. The resulting fits are shown in Fig. 2 of the main text. While the quality of the fits are less good than they are for the bulk, Fig.S3, they are sufficient for producing reliable trends in terms of the phases expected as a function of the on-site interaction U. Our thin film calculations start with 21 randomly generated coupling constants $m^i_{\alpha\beta} = \langle \hat{c}^{i\dagger}_\alpha \hat{c}^i_\beta \rangle$ per site, which are then iterated to convergence with a difference between $m^i_{\alpha\beta}$ input and output of less than 10^{-8}. The lattice size is selected by be 90 by 90 or 120 by 120. Around fifty sets of initial guesses are taken, and about two-thirds of them converge for most U values. The final convergent configuration with the minimum total energy is selected.

The magnetic moments are calculated in a way similar to the determination of the Landé-g factor. The g factor is obtained by

$$g\langle \mathbf{j}^2 \rangle = \langle (-g_l \mathbf{l} + g_s \mathbf{s}) \cdot \mathbf{j} \rangle, \tag{S2}$$

where $\mathbf{j} = -\mathbf{l} + \mathbf{s}$ is the total angular momentum in *local* coordinates. Here $-\mathbf{l}$ is the effective orbital angular momentum in t_{2g} subspace obtained by $P_{t_{2g}} \mathbf{L} P_{t_{2g}} = -\mathbf{l}_{l=1}$, where $P_{t_{2g}}$ is the projection operation into the t_{2g} subspace, and $g_l = 1, g_s = 2$. Then the total magnetic moment is determined by

$$\mathbf{m} = g\mu_B \langle \mathbf{j} \rangle. \tag{S3}$$

Most of the magnetic moment originates in the $j = 1/2$ subspace. Similar to the situation reported in bulk $Y_2Ir_2O_7$[16], we find an energy difference (around 1-150 meV/site, depending on the distance from the magnetic phase transition) between states with different order. All of our time-reversal symmetry broken solutions have non-zero net magnetization.

To compute the Z_2 invariant in the non-magnetic insulators, we use the formulation of Ref.[17]. We compute the Chern number in the magnetic insulating phases in a similar way[18].

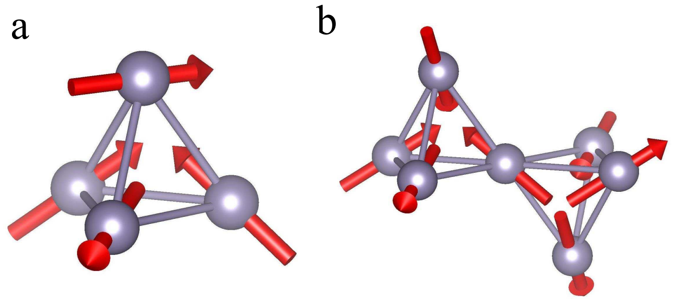

FIG. S5. The magnetic configurations at large U. (a) The magnetic configuration when $U = 1.30$eV in bilayer thin film. (b) The magnetic configuration when $U = 1.20$eV in TKT thin film. Both of them are in the magnetic insulating phase. In both (a) and (b), all the three in-plane ions in one unitcell possess magnetic moments with the same magnitude.

The magnetic metallic phase is close to the nonmagnetic to magnetic phase transition point, so the magnetic moments may have different configurations with very close energy. However, for a magnetic insulating phase with large U, the magnetic configuration is rather stable, and has the pattern shown in Fig.S5.

---

[1] P. Blaha, K. Schwarz, G. K. H. Madsen, D. Kvasnicka, and J. Luitz, *WIEN2K, An Augmented Plane Wave + Local Orbitals Program for Calculating Crystal Properties* (Karlheinz Schwarz, Techn. Universität Wien, Austria, 2001).
[2] P. Giannozzi, S. Baroni, N. Bonini, *et al.*, J. Phys.: Condens. Matter **21**, 395502 (2009).
[3] Z. Wu and R. E. Cohen, Phys. Rev. B **73**, 235116 (2006).
[4] N. Taira, M. Wakeshima, and Y. Hinatsu, J. Phys.: Condens. Matter **13**, 5527 (2001).
[5] A. Rüegg, C. Mitra, A. A. Demkov, and G. A. Fiete, Phys. Rev. B **85**, 245131 (2012).
[6] A. Rüegg, C. Mitra, A. A. Demkov, and G. A. Fiete, Phys. Rev. B **88**, 115146 (2013).
[7] J. C. Slater and G. F. Koster, Phys. Rev. **94**, 1498 (1954).
[8] A. A. Mostofi, J. R. Yates, Y.-S. Lee, I. Souza, D. Vanderbilt, and N. Marzari, Comput. Phys. Commun. **178**, 685 (2008).
[9] B.-J. Yang and N. Nagaosa, Phys. Rev. Lett. **112**, 246402 (2014).
[10] D. Pesin and L. Balents, Nat. Phys. **6**, 376 (2010).
[11] J. Kune, R. Arita, P. Wissgott, A. Toschi, H. Ikeda, and K. Held, Comput. Phys. Commun. **181**, 1888 (2010).
[12] J. Rieken, I. Anderson, and M. Kramer, *Innovative Powder Processing of Oxide Dispersion Strengthened ODS Ferritic Stainless Steels*, Tech. Rep. (Ames Laboratory (AMES), Ames, IA (United States), 2011).
[13] S. R. Bahn and K. W. Jacobsen, Comput. Sci. Eng. **4**, 56 (2002).
[14] T. Takeda, Z. Phys. B Con. Mat. **32**, 43 (1978).
[15] H. J. Monkhorst and J. D. Pack, Phys. Rev. B **13**, 5188 (1976).
[16] X. Wan, A. M. Turner, A. Vishwanath, and S. Y. Savrasov, Phys. Rev. B **83**, 205101 (2011).
[17] T. Fukui and Y. Hatsugai, J. Phys. Soc. Jap. **76**, 053702 (2007).
[18] T. Fukui, Y. Hatsugai, and H. Suzuki, J. Phys. Soc. Jap. **74**, 1674 (2005).
